# Effect of Hydroxypropylation on Functional Properties of Different Cultivars of Sweet Potato Starch in Sri Lanka

**DOI:** 10.1155/2014/148982

**Published:** 2014-08-31

**Authors:** Suraji Senanayake, Anil Gunaratne, K. K. D. S. Ranaweera, Arthur Bamunuarachchi

**Affiliations:** ^1^Department of Food Science & Technology, University of Sri Jayewardenepura, Sri Lanka; ^2^Faculty of Agricultural Sciences, Sabaragamuwa University of Sri Lanka, Belihuloya, Sri Lanka

## Abstract

Starches obtained from different cultivars of sweet potatoes commonly consumed in Sri Lanka, were chemically modified with hydroxypropyl substitution, to analyze the changes in the physicochemical properties. Significant changes (*P* < 0.05) in the crude digestibility level, thermal properties, and the water separation (syneresis) of starch gels (7.0% db) during cold and frozen storage were observed due to the modification. Hydroxypropylation increased the gel stability, water solubility, digestibility, and storage stability of the native starches in the cold storage to a significant level. Lowered gelatinization and retrogradation enthalpies as well as gelatinization temperature were observed for derivatized starches compared to the native starch. Low levels of pasting stability with increased levels of breakdown and reduced cold paste viscosity were observed in the hydroxypropylated starch samples except for the Malaysian cultivar (S5). Chemically modified starch gels stored under cold storage did not show a syneresis for two weeks in the cycle and the frozen storage showed much improved stability in the starch gels within the four-week cycle. Chemical modification of sweet potato starch with hydroxyl propyl substitution can enhance the functional characteristics of the native starch which will improve its potential application in the food industry.

## 1. Introduction

Starches have a wide spectrum of applications both in food and other industries such as textile and cosmetics. Native starches have restricted usage in food processing operations, distribution, and storage conditions due to the unfavourable characteristics prevailing. Native starches which have been modified either by physical or chemical treatments show much improved functional attributes that will have a broader area of usage in food processing operations [1]. Chemically modified food grade starches show increased levels of starch paste consistency, smoothness, paste clarity, and cold storage and freeze-thaw stability [2–4].

Rheological, morphological, and physicochemical characteristics can be improved through the chemical modification of the native starch by cross-linking, substitution, or reacting with acids or alkali. The amount of the chemical reagent required to achieve the functional properties needed in the starch may vary depending on the starch source, reagent type required for the substitution, the degree of substitution of the chemical reagent on the starch source, and the required range of properties in the final modified starch product [5, 6]. The changes occur in the starch structure and the regions containing the substituted groups can be detected through the scanning electron microscopy (SEM), colourimetrically or by nuclear magnetic resonance (NMR) [7].

Hydroxypropylation (HP) is commonly used in the chemical modification of the native starch due to much improved thermal characteristics upon gelatinization. Hydroxypropyl groups are being substituted to the native starch in the presence of alkali catalysts and the monosubstitution group in HP of the food grade starch should not exceed the 10% level [8]. It was reported by [9] that the chemical cross-linking of POCl_3_ in sweet potato starch mostly took place within the central regions of the starch granules and substitution taking place throughout the granules was observed by [10]. Occurrence of the initial derivatization in the more accessible amorphous regions followed by gradual advancement towards the more organized crystallite regions was observed by [11]. Substitution of the HP groups may enhance the free movement of the starch chains within the amorphous regions in the granule, due to the disruptions occurring among the inter- and intramolecular hydrogen bonds. The weakened internal bond structure in the starch granules due to the derivatized HP groups enhances the functional characteristics of the starch, such as freeze-thaw stability, reduced gelatinization temperature, high levels of peak viscosity, and starch paste clarity [12, 13].

The main source of the modified starch used in the food industrial applications in Sri Lanka is the corn starch and the country has to incur more expenditure in importing the product. Therefore it is important to utilize the alternative products which are feasible to manufacture locally with little or no modifications to improve the economic status of the country. Previous studies done by us using these commonly consumed sweet potato cultivars in Sri Lanka showed much favourable functional characteristics both in the native form as well as the physically modified form due to hydrothermal treatment [14]. Current study aimed at investigating the effectiveness of the chemical modification through HP substitution, to the same sweet potato cultivars in the improvement of the functional characteristics, which may enhance the potential application in the food industry.

## 2. Materials and Methods

### 2.1. Materials

Matured roots of sweet potatoes after about 2-3 days of harvesting were randomly selected from three different locations in Dhambulla, Gokarella, and Horana areas in Sri Lanka. Extracted starches from S1 (Wariyapola red), S3 (Wariyapola white), S4 (Pallepola variety), S5 (Malaysian variety), and S7 (CARI 273) cultivars were analysed for functional characteristics.

### 2.2. Starch Extraction

Starch separation was carried out according to the method described by [14]. Fresh roots were washed, peeled, diced, and wet milled at low speed in a laboratory scale blender with 1 : 2 (w/v) of tap water for 2 minutes and filtered through a gauze cloth. Residue was repeatedly wet milled and filtered thrice, and the suspension was kept overnight for the settling of starch. The supernatant was decanted and the settled residue was further purified with repeated suspension in tap water (1 : 2 w/v) followed by the settling for 3 h. The purified starch was dried at 35°C for 30 h in a forced air oven, sifted through 300 *µ*m sieve, sealed, and packed for analysis.

### 2.3. Hydroxypropylation

HP groups were substituted to the native starch according to the method described by [15]. A starch sample of 50 g/db, 110 mL of distilled water, and 10 g of Na_2_SO_4_ was mixed in a centrifuge tube. The pH was adjusted to 11.3 with 1 M NaOH and 4.5 mL of 3.0% propylene oxide was added for the substitution since previous studies have shown more effective substitution levels at this level [15]. Capped the sample tube immediately and shaken vigorously for proper mixing. Then incubated at 35°C in a shaking water bath for 24 h and the reaction was terminated by adjusting the pH to 5.3 with 1 M HCl. The slurry was then centrifuged at 3000 ×g for 10 mins and the remaining residue after discarding the supernatant was washed with distilled water and dried at 35°C.

### 2.4. Swelling Power (SP) and Water Soluble Index (WSI)

Swelling power and the WSI of the native and the chemically modified starches were determined by the method described by [16]. Weighed 100 mg/db starch into a screw-cap test tube and added 10 mL distilled water. Tightly capped tubes were placed on a vortex mixer for 10 seconds for homogeneous mixing. Samples were then heated at 85°C in a water bath for 30 mins with occasional mixing and immediately cooled to ambient temperature. The samples were centrifuged at 2000 ×g for 30 mins, and the remaining residue in the tube was weighed (*W*_s_). The supernatant was dried until a constant weight (*W*_1_) was obtained in a drying oven at 100°C. The SP and WSI were calculated as follows:

(1) $$\begin{array}{c} \text{SP}=\frac{W_{\text{s}}}{\left[0.1\times \left(100\%-\text{WSI}\right)\right]\;}\left(\frac{\text{g}}{\text{g}}\right), \end{array}$$

where WSI = *W*_1_/0.1 × 100%.

### 2.5. Gelatinization

The thermal characteristics of starch gelatinization and retrogradation were determined by using differential scanning calorimeter (DSC) (TA 2920, Newcastle, DE). Approximately 3 mg of starch was mixed with 9 *µ*L distilled water on the aluminum DSC pan and kept at ambient temperature for 1 h to equilibrate the mixture. The DSC pan was scanned from 30 to 120°C at a rate of 10°C/min. An empty pan was kept as a reference and all the measurements were performed in triplicate. The corresponding enthalpy (J/g) was expressed on the dry weight basis of the starch.

### 2.6. Retrogradation

A starch gel was prepared by using a ratio of 1 : 1 starch to water in the DSC pan and equilibrated for 1 h. After the equilibration the DSC pan was heated using a convection oven at 120°C for 10 mins to gelatinize the starch. The DSC pan with the gelatinized starch was stored at 4°C for 24 h to initiate the process of nucleation. Prior to scanning by the DSC, the sample was kept at 40°C for 7 mins. The DSC pan was heated from 30 to 120°C at a rate of 10°C/min. Change in Δ*H*_*R*_ (J/g) was measured for triplicated samples by keeping an empty pan as a reference.

### 2.7. Pasting Properties

Pasting properties of the native and the substituted starches were determined by using a rapid visco analyzer (3D, Newport Scientific, Warriewood, Australia). A starch sample with approximate weight of 2.0 g/db was mixed with 25.5 g of distilled water in the RVA canister to a total sample weight of 27.5 g (8.1% starch concentration). The total pasting cycle was set for 22 mins; the homogenized slurry was heated at 50°C for 1 min and then at 95°C for 12.5 mins. Then the sample was cooled to 50°C within 7.5 mins and held at 50°C for 1 min to complete the cycle. Triplicate measurements were taken for all the samples.

### 2.8. Digestibility

*In vitro* starch digestibility of native and HP starches was measured by using the method described by [17]. A starch sample of 500 mg was placed in a 50 mL centrifuge tube and 15 mL phosphate buffer (0.15 M, pH 6.5), 30 mg CaCl_2_, 30 mg gelatin, and 30 mg pancreatin were added (Sigma Aldrich, USA). The capped tubes were kept at 37°C in a shaking water bath for 12 h and the reaction was terminated by adding 5 mL of 1% H_2_SO_4_. Digested suspension was centrifuged at 20,000 ×g for 10 mins and the supernatant was decanted. The remaining residue pellet was dispersed with 15 mL of 80% ethanol and recentrifuged for 5 mins. The resulting pellet was dried at 50°C for 6 h and then at 80°C to a constant weight. Sample was weighed at the ambient temperature and the percentage weight loss after the* in vitro* digestion was considered as the crude digestibility level of the starch. A sample blank without pancreatin was kept as a control to adjust the results.

### 2.9. Syneresis of Cooked Starch after Refrigeration and Freeze-Thaw

Syneresis of starch gels of native and modified starches during cold storage and frozen storage was determined with slight modifications to the methods described by [17, 18]. A starch suspension (7% dry basis, w/w) was prepared with 0.1% sodium benzoate to prevent microbial spoilage during repeated refrigeration or freeze-thaw treatments. The suspension was adjusted to pH 6.5 with 0.01 M NaOH or HCl solutions and heated at 92.5°C for 30 mins. After cooling to ambient temperature, 10 g of paste was transferred into polypropylene tubes and capped. The starch pastes were stored at 4°C for 1 week and then held at ambient temperature for 6 h and centrifuged at 300 ×g for 10 min. The supernatant was decanted, weighed, and calculated as a percentage from the original weight. This refrigeration cycle was repeated four times for all samples.

Starch pastes were stored in the freezer (−18°C) for 1 week and then thawed at 40°C for 2 h to determine the freeze-thaw stability. The thawed samples were centrifuged at 3000 ×g for 10 mins and the residue was weighed after discarding the supernatant. The gels were refrozen following the measurement of expelled water to repeat the cycle. All starch samples were subjected to four freeze-thaw cycles. Cumulative values for expelled water were obtained after calculating each percentage of expelled water from the remaining gel. Total syneresis was calculated by the addition of mean values of the expelled water from the triplicated samples, after each refrigeration or freeze-thaw treatment.

### 2.10. Statistical Analysis

All numerical results were averages of three independent replicates. Data were analysed by one-way analysis of variance (ANOVA) using Minitab Ver 14.0. The statistical significance was determined by using Tukey's test (*P* < 0.05).

## 3. Results and Discussion

### 3.1. Swelling Power, Water Soluble Index, and Digestibility

Substituted starches with HP groups showed significantly high level (*P* ≤ 0.05) of swelling power and water soluble index compared to their native forms in all the cultivars studied. No significant difference in SP and WSI were observed in the chemically modified S1, S3, and S5 starch types (Table 1). Comparably high amount of swelling than the native starch was observed in the S7 starch due to the chemical modification. The degree of HP substitution studies showed that the HP substituents were not distributed evenly over the starch chains of sweet potato and about 2 hydroxypropyl groups per 10 glucose units and it was hypothesized that a higher level of substitution would take place in the amorphous regions and the peripheral regions of the starch clusters [19]. It was reported [20] that the swelling power and water solubility increase due to hydroxypropylation and high level of molecular substitution of HP groups greatly influences these values.

Loosened internal structure of the starch due to the substituted HP groups, which are hydrophilic in nature, enhances the attraction of more water molecules into the rearranged granular structure that causes early swelling in the granule with an increased level. High level of swelling promotes rapid granular rupture which causes an increased level of amylose leaching that will have a positive impact on the water solubility [15]. The increased pH due to alkaline treatment during hydroxypropylation ionizes the hydroxyl groups in starch chains, thus disrupting the hydrogen bonds within the molecules, which help in increase of the granular swelling [11, 15]. It was also observed by [15] that the existence of amylose-lipid complex at higher pH levels can also bring about higher values in swelling.

The digestibility level of the native starches was within the range of 19.3%–23.5% and the range increased up to 20.6–47.3% when derivatized with HP groups. Comparably no significant difference (*P* ≤ 0.05) was observed in the digestibility of native and hydroxypropylated S7 starch. This may be due to the lower degree of substitution of HP groups into the granular matrix than the other chemically modified starch types and the resulting crystallite structure may have not influenced the enzyme attack in the S7 starch type. Initial X-ray diffraction study prior to the treatment and after together with analysis of the degree of substitution would give more evidence in this. S3, S4, and S5 starches showed nearly twofold increases in digestibility due to derivatization (Table 1). Previous studies of flour digestibility of these cultivars with pancreatin enzyme were within the range of 36–55% and the lowest and the highest values were shown in S1 and S7 cultivars, respectively [14]. A wide variation in digestibility was reported by [18] among different cultivars of sweet potatoes. It was reported by [21, 22] that raw starch had poor digestibility around 24% with α-amylase enzyme which was compatible with our findings.

Our results showed that hydroxypropylation has significantly increased the digestibility level of native starches in all the cultivars except for the S7. Starches of sweet potatoes are found to be having specific susceptible zones to α-amylase [23] and the regions that are susceptible to enzyme attack are mostly the amorphous regions of the granule. Alteration of the crystalline structure of the granule by derivatization of hydroxypropyl groups may have influenced susceptibility for enzymatic degradation. The weakened starch molecular structure due to rearrangement resulted from HP substitution may have widened the surface channels to facilitate the entrance for more enzymes resulting improved levels of* in vitro* digestibility [17].

### 3.2. Gelatinization

The gelatinization temperature range (*T*_*c*_–*T*_*o*_) has changed from 15.5–17.1°C in the native starches to 13.9–16.8°C when substituted. Each starch type showed a reduced level of gelatinization temperature range than its native form due to chemical modification (Table 2) and the low onset temperature (*T*_*o*_) and conclusion temperature (*T*_*c*_) may due to the change in heterogeneity of the starch molecular structure due to the substitution. Significant reduction (*P* < 0.05) in the peak temperature (*T*_*p*_) of gelatinization was observed in all the chemically modified starches. Reduced values for the transition temperatures (*T*_*o*_, *T*_*p*_, and *T*_*c*_), and enthalpy (Δ*H*) of gelatinization for HP starches and further reduction of the values with increased levels of molecular substitution were reported by [24].

**Figure 1:**
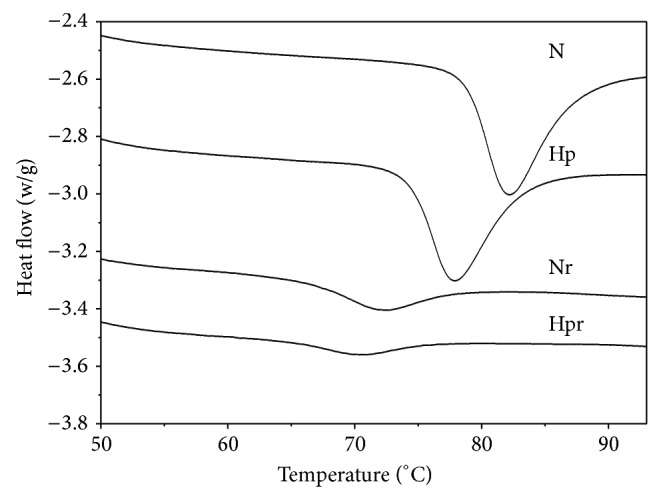
Representative DSC curves of the native and hydroxypropylated starch. Gelatinization of the native starch (N), hydroxypropylated starch (Hp), retrogradation of native starch (Nr), and retrogradation of hydroxypropylated (Hpr) sweet potato starch (S1).

Hydrophilic nature of the derivatized groups attract more water and early swelling due to the rearrangement of starch granules may have reduced the peak temperature and the melting enthalpy of gelatinization (Δ*H*_*g*_) in the HP starches (Figure 1). Significantly high reduction in energy requirement for gelatinization was observed in substituted S7 cultivar compared to the other cultivars and there was no significant difference in the enthalpy reduction in the chemically modified other starch types (Table 2). Degree of substitution analysis of each type would give a more comprehensive picture of the enthalpy changes.

During the substitution process, reagent solution readily enters the amorphous regions of the granules and derivatizes them first, which make way to further swelling of the granule and enhance the exposure of the crystalline regions with the reagent solution [11]. More derivatization could disrupt more hydrogen bonds in the crystalline region and reduce the energy requirement for gelatinization of the crystalline region. Derivatization of sweet potato starch with phosphorus oxychloride was shown to be having a negative impact on reducing the gelatinization temperature and enthalpy [9]. This trend was also reported with acetylated oat and wheat starches [24, 25]. Significant reduction of gelatinization temperature and enthalpy in alkaline treated and HP wheat, potato and waxy maize starch was reported by [15]. Our results clearly showed the positive impact of derivatization of native starches with HP groups in reducing the gelatinization temperature and the melting energy for gelatinization.

### 3.3. Retrogradation

HP substitution has significantly reduced the amylopectin retrogradation in all the starches. Amylopectin chains with substituted HP groups could reduce the extent of reassociation of amylopectin molecules or retrogradation, by inhibiting the interchain association. Less stable starch crystallites due to restricted reassociation have lowered the melting enthalpy of retrogradation (Δ*H*_*R*_). Melting enthalpy of retrogradated amylopectin ranged from 3.5 to 5.8 J/g and from 1.3 to 2.5 J/g in native starches and the HP starches, respectively (Table 3). S5 showed lower melting enthalpy both in the native and the derivatized form compared to other cultivar types and S3 and S7 had comparatively low levels of retrogradation due to the chemical modification. Highly derivatized amylopectin could reduce the reassociation between the neighboring amylopectin molecules to a greater extent and result in decreasing the amylopectin retrogradation in the starch pastes during storage. Also the water molecules bound to the hydrophilic substituted groups retard the water separation from the gel matrix and ensure the crystallite perfection within the starch granules.

### 3.4. Pasting Properties

**Figure 2:**
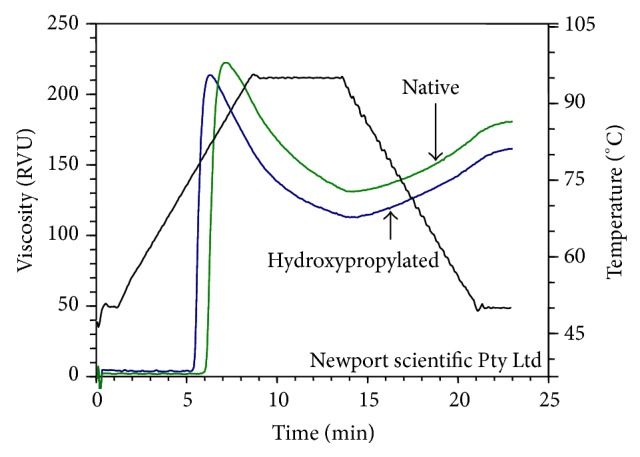
Representative RVA curves of native and hydroxypropylated sweet potato starch (S1).

Pasting can be defined as series of processes that take place following the gelatinization, the dissolution of starch due to granular swelling, release of granular components gradually, and total disruption of the granules. When gelatinized starch paste is subjected to cooling the rapid reassociation of linear amylose through forming a gel matrix governing the extent of viscosity increases in starch pastes. Representing of RVA curve for our analysis is given in Figure 2 and the substituted starches showed an onset of early pasting. The reduced level of reassociation of linear amylose molecules due to derivatization, with HP groups, may have caused the reduction in the cold paste viscosity (CPV) and hot paste viscosity (HPV) in S1, S3, S4, and S7 starches. There was no significant difference (*P* < 0.05) in the cold paste viscosity (CPV) of the substituted and the native S5 starch.

High level of paste stability was observed in chemically modified S5 starch compared to other substituted starch types. Significant increase in the peak viscosity was observed in the HP substituted S7 starch (Table 4). Reduced level of breakdown (BD) and increased setback (SB) in the HP substituted S5 starch showed high level of tolerance to heat and shear stress and low level of retrogradation when cooling, respectively (Tables 3 and 4). High level of BD and the low levels of PV in the chemically modified S1, S3, S4, and S7 starches may have resulted from the rapid disintegration of the leached amylose from the weakened granular structure due to the substitution at higher temperatures. The structural changes at elevated temperatures result in rapid loss in viscosity and the high level of pasting stability of the native starches may have resulted due to the nondisruption of the granules during the pasting process compared to the substituted starch. No significant change in the SB was observed in the native and substituted forms of S1 and S3 starches. Overall pasting results showed no significant effect of hydroxypropylation on native starch except for the cultivar S5, in improving the pasting characteristics.

### 3.5. Syneresis

#### 3.5.1. Syneresis during Refrigeration

HP has greatly influenced the reduction of syneresis in the native starch during the four-week refrigeration cycle. More than tenfold increase in the water loss (%) was observed in the native starch during this period and a significantly high level of water loss (*P* < 0.05) was shown in the native starch gels of S4 and S7 (Table 5). Chemical modification has completely prevented the water loss within the first two weeks of the storage, and a sharp increase in the syneresis was observed during the third week of the storage in the substituted starches. Nonappearance of expelled water in the modified starches within the first two weeks of the cycle may due to the tightly bound nature of the gel matrix with the water molecules due to the hydrophilicity of the derivatized HP groups. Initiation of starch nuclei formation in the starch crystallization may have also occurred during this period and the rapid increase in syneresis within the third week of the cycle would have been caused by the increased levels of water release due to crystallite perfection. Significantly low levels (*P* < 0.05) of water release from the gel matrixes of the derivatized starches during the fourth week may have been caused by the retardation of the retrogradation process due to the high affinity to water by the HP groups. Inconsistent pattern of water loss shown by the native starches during the four-week refrigeration cycle may be due to the comparably free formation of starch nuclei for crystallization caused by the noninterruption by the HP groups.

#### 3.5.2. Syneresis during Freeze-Thaw Cycles

**Figure 3:**
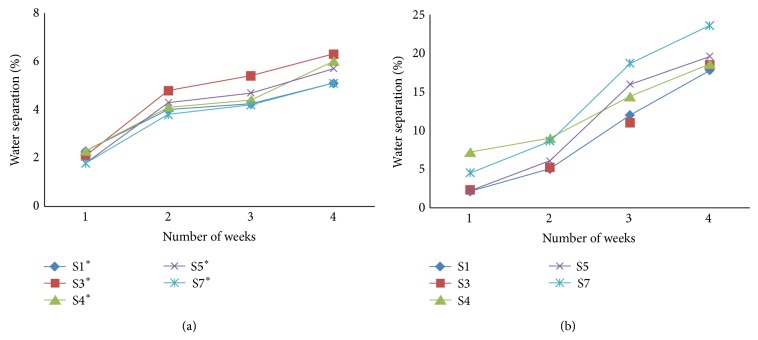
Percentage of syneresis during four freeze-thaw cycles. ^*^Hydroxypropylated starch (water separation of hydroxypropylated starch (a) and water separation of native starch (b)).

Significantly low level of water loss was observed during the four-week freeze thaw cycle of the chemically modified starches compared to the native forms (Figure 3). Comparatively high amount of water loss was observed in the substituted starches between the first and the second weeks of the cycle. This may have occurred due to the rapid formation of starch crystallites caused by increased amount of nuclei formation and releasing more water as a result. The occurrence of crystallite formation may have reduced within the next few weeks due to the low temperature storage. Slow rate of crystallite formation together with tightly bound water molecules to the HP substituted starch gel matrix may have caused the low level of water separation within the third and the fourth weeks of the cycle in the chemically modified starches. Similar phenomena can be used to explain the rapid increase in the syneresis (%) within the second and third weeks of the native starches (Figure 3).

Starch rich regions will be created in a gel matrix when frozen, where water molecules form ice crystals through coagulation by forming a separate phase. Resulting high concentrations of starch chains reassociate by forming thick filaments. Upon thawing, ice phase transmission to water leaves a sponge like starch paste [24]. Similar to cold storage, derivatized frozen starch showed low levels of syneresis compared to the native starches and there was no significant difference (*P* < 0.05) in the syneresis (%) of S1, S4, S5, and S7 and S3 showed a slightly high level of expelled water compared to the other types. As observed in the cold storage, native forms of the S7 showed a high level of syneresis and a low level of water loss in the S3 cultivar (Figure 3).

Syneresis in the native forms of the refrigerated samples was significantly higher than the frozen samples, but this feature was not evident in the chemically modified samples. A wide range in the levels of expelled water values during refrigeration and high levels of absorbed water values during FT cycles in native root starches were reported by [18]. About 71.3% of water loss in native starch gels (7.0% db, w/w), within three FT cycles, was reported by [20]. Low levels of syneresis and increased freeze-thaw stability due to HP in native starches were also reported [20]. Overall results showed a significantly high level of starch gel stability, both in the refrigerated and frozen storage of HP substituted starch, which can be used in food products with extended periods of shelf life during the refrigerated and frozen storage.

## 4. Conclusions

Hydroxypropyl substitution to native starches significantly increased (*P* < 0.05) the* in vitro* digestibility level, swelling power, water solubility, and starch gel stability during the frozen and the refrigerated storage. Chemically modified starches showed lower gelatinization temperatures and no significant effect on the pasting characteristics was observed except for the S5 starch. Chemical modification has significantly reduced the retrogradation levels in S3 and S7 types while preventing the syneresis in S4 type during the cold storage. Significant level of increase in the percentage digestibility, SP, WSI, paste stability due to high SB, CPV, and low BD and also the increased level of stability during the refrigerated and frozen storage were observed in S5 cultivar due to HP substitution. With much improved functional characteristics due to chemical modification with substituted HP groups, tested starches showed a high potential in being used as a food ingredient in the industry and the characterization of these starches with different levels of HP should be tested to achieve the desired level of pasting properties.

## Figures and Tables

**Table 1:** Variation in digestibility, SP, and WSI in native starch and ^*^hydroxypropylated starch.

Sample	Digestibility (%)	SP (g/g)	WSI (%)
S1	21.7 ± 0.2^d^	7.9 ± 0.1^c^	1.6 ± 0.1^c^
S1∗	30.9 ± 0.7^c^	9.6 ± 0.5^b^	4.2 ± 0.3^a^
S3	21.9 ± 1.5^d^	8.7 ± 0.2^c^	1.8 ± 0.05^c^
S3∗	40.6 ± 1.6^b^	9.4 ± 0.1^b^	4.4 ± 0.3^a^
S4	23.5 ± 0.5^d^	8.7 ± 0.1^c^	1.3 ± 0.05^c^
S4∗	47.3 ± 0.7^a^	12.3 ± 0.1^a^	3.2 ±0.02^b^
S5	23.3 ± 0.1^d^	8.0 ± 0.1^c^	2.1 ± 0.05^c^
S5∗	41.8 ± 0.5^b^	9.5 ± 0.2^b^	4.6 ± 0.04^a^
S7	19.3 ± 0.3^d^	5.8 ± 0.1^d^	0.5 ± 0.01^d^
S7∗	20.6 ± 0.5^d^	9.8 ± 0.1^b^	2.6 ± 0.02^b^

Values denoted by similar superscripts in each column have no significant difference at *P* > 0.05 level.

**Table 2:** Gelatinization parameters^x^ of native and hydroxypropylated^*^ sweet potato starch.

Cultivar	*T*_*o*_ (°C)	*T*_*p*_ (°C)	*T*_*c*_ (°C)	*T*_*r*_ (°C)	Δ*H*_*g*_ (J/g)
S1	78.3 ± 1.2^a^	82.0 ± 0.7^a^	94.0 ± 1.1^a^	15.7 ± 0.1^b^	15.7 ± 0.4
S1∗	74.2 ± 0.5^b^	77.8 ± 0.2^b^	88.1 ± 1.8^c^	13.9 ± 0.4^c^	13.4 ± 0.7
S3	77.6 ± 0.4^a^	81.9 ± 0.5^a^	94.5 ± 1.4^a^	16.9 ± 0.1^a^	16.4 ± 0.7
S3∗	73.8 ± 0.7^b^	78.1 ± 0.6^b^	67.8 ± 1.8^c^	14.0 ± 0.3^c^	13.9 ± 0.2
S4	77.0 ± 0.3^a^	81.8 ± 0.3^a^	94.3 ± 0.9^a^	17.3 ± 0.2^a^	14.3 ± 0.3
S4∗	71.9 ± 0.4^c^	77.2 ± 0.4^c^	88.1 ± 1.3^c^	16.2 ± 0.2^a^	14.3 ± 0.3
S5	77.3 ± 0.6^a^	80.5 ± 0.2^a^	92.8 ± 1.5^b^	15.5 ± 0.2^b^	15.5 ± 0.6
S5∗	73.3 ± 0.6^b^	76.8 ± 0.2^c^	87.5 ± 2.1^c^	14.2 ± 0.3^c^	13.0 ± 0.4
S7	78.6 ± 0.4^a^	83.6 ± 0.2^a^	95.7 ± 1.8^a^	17.1 ± 0.1^a^	20.1 ± 0.5
S7∗	73.8 ± 0.7^b^	79.4 ± 0.5^b^	90.6 ± 2.0^c^	16.8 ± 0.4^a^	15.2 ± 0.3

^x^ *T*_*o*_ = onset temperature; *T*_*p*_ = peak temperature, *T*_*c*_ = conclusion temperature; *T*_*r*_ = temperature range of gelatinization; Δ*H*_*g*_ = gelatinization enthalpy.

Values denoted by different superscripts in each column show significant difference at *P* < 0.05.

**Table 3:** Retrogradation^a^ of native and hydroxypropylated sweet potato starch as measured by DSC.

Cultivar	Treatment	Δ*H*_*R*_ (J/g)
S1	Native Hydroxypropylated	3.9 ± 0.5^f^ 1.7 ± 0.4^i^
S3	Native Hydroxypropylated	5.3 ± 0.3^e^ 1.6 ± 0.3^i^
S4	Native Hydroxypropylated	5.4 ± 0.1^e^ 2.5 ± 0.1^h^
S5	Native Hydroxypropylated	3.5 ± 0.4^g^ 1.3 ± 0.2^j^
S7	Native Hydroxypropylated	5.8 ± 0.4^e^ 2.1 ± 0.4^h^

^a^Δ*H*_*R*_ = melting enthalpy of retrograded amylopectin.

^b^Values are means of triplicate determinations ± standard deviation.

^c^Different superscripts in the column show significant difference at *P* < 0.05 level.

**Table 4:** Pasting properties^x^of native and hydroxypropylated^*^ sweet potato starch.

Cultivar	PV	HPV	BD	CPV	SB
S1	222 ± 4.5^b^	131 ± 5.9^c^	91 ± 5.3^c^	180 ± 6.1^d^	49 ± 2.4^e^
S1∗	217 ± 1.5^c^	114 ± 2.8^d^	103 ± 4.6^b^	163 ± 3.5^e^	49 ± 1.5^e^
S3	225 ± 2.1^b^	145 ± 6.1^b^	79 ± 3.6^c^	208 ± 2.8^b^	62 ± 1.8^c^
S3∗	202 ± 3.2^d^	116 ± 5.1^d^	86 ± 2.1^d^	179 ± 2.6^d^	63 ± 1.5^c^
S4	257 ± 4.2^a^	162 ± 3.2^a^	95 ± 4.2^c^	251 ± 3.2^a^	89 ± 5.2^a^
S4∗	209 ± 2.1^d^	105 ± 4.1^e^	104 ± 3.5^b^	164 ± 2.5^e^	58 ± 2.1^d^
S5	248 ± 3.2^a^	129 ± 4.2^c^	118 ± 2.9^a^	178 ± 2.6^d^	48 ± 4.1^e^
S5∗	191 ± 2.5^e^	110 ± 3.8^e^	81 ± 4.1^d^	172 ± 2.5^d^	62 ± 3.2^c^
S7	214 ± 4.1^c^	141 ± 3.1^b^	73 ± 6.3^e^	212 ± 3.2^b^	71 ± 3.2^b^
S7∗	247 ± 3.1^a^	122 ± 2.5^c^	125 ± 2.9^a^	189 ± 2.5^c^	67 ± 2.7^c^

^x^PV = peak viscosity; HPV = hot paste viscosity; BD = breakdown; CPV = cold paste viscosity; SB = setback. Values are the means of triplicate determinations ± standard deviation. Different superscripts in each column are significantly different at *P* < 0.05 level.

**Table 5:** Syneresis (%) of starch gels within four week refrigerated storage.

Cultivar type	1st week	2nd week	3rd week	4th week
S1	2.9 ± 0.1^d^	11.9 ± 0.1^a^	10.5 ± 0.1^b^	10.8 ± 0.1^a^
S3	3.6 ± 0.1^c^	9.3 ± 0.1^d^	8.4 ± 0.1^d^	9.4 ± 0.2^c^
S4	20.0 ± 0.2^a^	7.2 ± 0.2^e^	17.1 ± 0.3^a^	10.0 ± 0.1^b^
S5	2.0 ± 0.1^e^	9.7 ± 0.2^c^	9.7 ± 0.1^c^	11.2 ± 0.2^a^
S7	11.6 ± 0.1^b^	10.0 ± 0.1^b^	8.8 ± 0.2^d^	8.7 ± 0.3^d^
S1∗	0.0	0.0	6.3 ± 0.1^e^	0.9 ± 0.1^f^
S3∗	0.0	0.0	5.9 ± 0.2^f^	1.2 ± 0.1^ef^
S4∗	0.0	0.0	6.1 ± 0.1^ef^	1.6 ± 0.2^e^
S5∗	0.0	0.0	6.5 ± 0.1^e^	0.7 ± 0.1^efg^
S7∗	0.0	0.0	5.7 ± 0.2^f^	1.0 ± 0.1^efg^

∗Hydroxypropylated starch. Each value represents mean of triplicates, and data with different superscripts in each column are different with statistical significance (*P* < 0.05).
